# The EGFR/ErbB3 Pathway Acts as a Compensatory Survival Mechanism upon c-Met Inhibition in Human c-Met^+^ Hepatocellular Carcinoma

**DOI:** 10.1371/journal.pone.0128159

**Published:** 2015-05-22

**Authors:** Steven N. Steinway, Hien Dang, Hanning You, C. Bart Rountree, Wei Ding

**Affiliations:** Department of Pediatrics, The Pennsylvania State University College of Medicine, Hershey, Pennsylvania, United States of America; University of Central Florida, UNITED STATES

## Abstract

**Background:**

c-Met, a high-affinity receptor for Hepatocyte Growth Factor (HGF), plays a critical role in tumor growth, invasion, and metastasis. Hepatocellular carcinoma (HCC) patients with activated HGF/c-Met signaling have a significantly worse prognosis. Targeted therapies using c-Met tyrosine kinase inhibitors are currently in clinical trials for HCC, although receptor tyrosine kinase inhibition in other cancers has demonstrated early success. Unfortunately, therapeutic effect is frequently not durable due to acquired resistance.

**Methods:**

We utilized the human MHCC97-H c-Met positive (c-Met^+^) HCC cell line to explore the compensatory survival mechanisms that are acquired after c-Met inhibition. MHCC97-H cells with stable c-Met knockdown (MHCC97-H c-Met KD cells) were generated using a c-Met shRNA vector with puromycin selection and stably transfected scrambled shRNA as a control. Gene expression profiling was conducted, and protein expression was analyzed to characterize MHCC97-H cells after blockade of the c-Met oncogene. A high-throughput siRNA screen was performed to find putative compensatory survival proteins, which could drive HCC growth in the absence of c-Met. Findings from this screen were validated through subsequent analyses.

**Results:**

We have previously demonstrated that treatment of MHCC97-H cells with a c-Met inhibitor, PHA665752, results in stasis of tumor growth *in vivo*. MHCC97-H c-Met KD cells demonstrate slower growth kinetics, similar to c-Met inhibitor treated tumors. Using gene expression profiling and siRNA screening against 873 kinases and phosphatases, we identified ErbB3 and TGF-α as compensatory survival factors that are upregulated after c-Met inhibition. Suppressing these factors in c-Met KD MHCC97-H cells suppresses tumor growth *in vitro*. In addition, we found that the PI3K/Akt signaling pathway serves as a negative feedback signal responsible for the ErbB3 upregulation after c-Met inhibition. Furthermore, *in vitro* studies demonstrate that combination therapy with PHA665752 and Gefitinib (an EGFR inhibitor) significantly reduced cell viability and increased apoptosis compared with either PHA665752 or Gefitinib treatment alone.

**Conclusion:**

c-Met inhibition monotherapy is not sufficient to eliminate c-Met^+^ HCC tumor growth. Inhibition of both c-Met and EGFR oncogenic pathways provides superior suppression of HCC tumor growth. Thus, combination of c-Met and EGFR inhibition may represent a superior therapeutic regimen for c-Met^+^ HCC.

## Introduction

Hepatocellular carcinoma (HCC) represents the third leading cause of cancer-related death worldwide, and HCC is the only carcinoma with increasing mortality in the United States during the last decade [1]. Although surgical resection and transplantation have significantly improved survival in patients with small tumors with no evidence of invasion or metastasis, the prognosis of HCC for late stage disease remains very poor [2]. In addition, within HCC transplant patients, recurrent and metastatic disease remain the most important factors for survival [3]. In addition to tumor number, size, and vascular invasion observed in imaging studies, a molecular characteristic that appears to predict poor survival in HCC is c-Met expression [4–7].

Hepatocyte Growth Factor (HGF) is produced by stromal cells. HGF acts on c-Met, a high affinity receptor tyrosine kinase [8]. Following c-Met phosphorylation and activation, multiple downstream targets, such as the PI3K/Akt and MAPK/Erk pathways, are activated [9–11]. Through these intermediary pathways, HGF-induced c-Met activation triggers a variety of cellular responses, including proliferation, survival, cytoskeletal rearrangements, cell-cell dissociation, and motility [8, 12]. Although HGF/c-Met signaling does not have a known role in liver homeostasis during normal physiologic conditions, many studies have demonstrated the important role of HGF/c-Met in liver regeneration, hepatocyte survival, and tissue remodeling after acute injury [13, 14].

Within cancer, the HGF/c-Met axis mediates a proliferative advantage and promotes tumor invasion and metastasis [8, 12, 15–17]. As a result of the strong clinical correlation between c-Met expression and metastatic disease, c-Met has been targeted therapeutically to suppress tumor growth and metastasis in lymphoma, gastric cancer, melanoma, and lung cancer [18, 19]. In murine models of liver cancer, c-Met expression correlated with aggressive, metastatic disease [20]. We have recently demonstrated that c-Met inhibition results in tumor stasis in c-Met^+^ tumors; however c-Met inhibition is unable to completely eradicate HCC [21]. We hypothesized that compensatory survival signals are activated by c-Met inhibition in c-Met^+^ HCC to drive tumor growth. The goal of our current study is to identify secondary therapeutic targets to use in combination with c-Met inhibition to more robustly suppress HCC growth and survival.

In the current study, we used high-throughput siRNA screening and microarray pathway analysis to identify putative compensatory survival proteins, which could drive c-Met^+^ HCC growth in the absence of c-Met. Our analyses identified the EGFR pathway as a compensatory survival pathway after c-Met inhibition in c-Met^+^ HCC. We specifically identified that EGFR receptor ErbB3 and ligand TNF-α are upregulated after c-Met pathway suppression and that combination therapy with c-Met and EGFR inhibitors is superior to c-Met monotherapy *in vitro*. The use of high throughput screening to identify a therapeutic combination that is superior to c-Met monotherapy makes this a novel and important translational HCC study.

## Materials and Methods

### Cell culture

The human HCC cell lines MHCC97-L and MHCC97-H [22, 23] were provided by Dr. Xinwei Wang, the National Cancer Institute (NCI), under agreement with Liver Cancer Institute, Zhongshan Hospital, Fudan University, Shanghai, China [24]. MHCC97-L and MHCC97-H cell lines were previously derived from the parental cell line MHCC97, with the purpose of having cells with different metastatic potential for the study of metastasis-related mechanisms. The two clones have high (MHCC97-H) and low (MHCC97-L) metastatic potential [23]. MHCC97-L and MHCC97-H cells were maintained in DMEM/High glucose medium (Hyclone Laboratories, South Logan, Utah) supplemented with 10% defined FBS (Hyclone Laboratories), 100 μg/ml penicillin and 100 μg/ml streptomycin. Cells were cultured in a humidified incubator with 5% CO_2_ at 37°C. The human HCC cell line Huh7 was provided by Dr. Jianming Hu, Penn State College of Medicine, Department of Microbiology and Immunology[25]. The human HCC cell line Hep3B was provided by Dr. Xin Chen, Department of Bioengineering and Therapeutic Sciences, University of California San Francisco [26]. Huh7 and Hep3B cells were maintained in DMEM/F12 medium supplemented with 10% FBS, 100 μg/ml penicillin and 100 μg/ml streptomycin. The human HCC cell line SNU-449 was acquired from the American Tissue Culture Collection (ATCC; Manassas, Virginia) and grown in RPMI medium supplemented with 10% FBS.

### shRNA plasmid constructs

TG320418 HuSH 29mer shRNA constructs against c-Met in pGFP-V-RS vector were purchased from OriGene (Rockville, MD). The following constructs have been validated using real-time PCR assays and have been used for developing stable c-Met knockdown cell lines. The c-Met shRNA targeting sequence: 5’-TACTGCTGACATACAGTCGGAGGTTCACT-3’. The scrambled shRNA construct with pGFP-V-RS backbone was purchased from OriGene (Cat# TR30013).

### Development of stable c-Met shRNA HCC cells

MHCC97-H cells were transfected with either a scrambled shRNA or c-Met shRNA plasmid using Fugene 6 transfection reagents (Promega, Madison, WI). 24 h after transfection, puromycin (2 g/ml) was added to select stable c-Met shRNA clones. Single clones of stable MHCC97-H cells transfected with either scrambled shRNA or c-Met shRNA were isolated and expanded, and knockdown of c-Met expression was validated using both real-time PCR and Immunoblot assays as previously described [6, 27].

### siRNA library screening

Invitrogen’s siRNA screening library covering 873 kinases and phosphatases was utilized to screen for targets responsible for bypass survival mechanisms after c-Met inhibition. 5×10^3^ MHCC97-H c-Met shRNA cells were plated in 96-well plates and reverse transfected (cells were added to 10 nM siRNA and 0.2 μl RNAiMAX pre-added to wells) with individual siRNA using lipid-mediated transfection with Lipofectamine RNAiMAX (Life Technologies Corporation, Grand Island, NY). 48 hours after transfection, cell viability was assessed using XTT (cell viability) assay, and siRNA that resulted in cell viability Z-score of -2 or less was further validated (2 standard deviations below the population mean).

### Cell viability assay

Cell viability was performed using an XTT [2,3-bis(2-methoxy-4-nitro-5- sulfophenyl)-2H-tetrazolium-5-carboxanilide] kit (Trevigen, Gaithersburg, MD) according to the manufacturer’s protocol as previously described [21].

### Immunoblot

Cell lysates were collected, and blotted as previously described [28]. c-Met (#8198), phospho-c-Met (Tyr1349; #3133), phospho-c-Met (Tyr1234/1235; #3077), Akt (#9272), phospho-Akt (Ser473; #9271), Erk1/Erk2 (#9107), phospho-Erk1/Erk2 (Thr202/204; #4376), EGFR (#2646), phospho-EGFR (Tyr1068; #3777), phosphor-EGFR (Tyr1173; #4407) ErbB3 (#4754), PARP (#9532) monoclonal antibodies were purchased from Cell Signaling Technology (Danvers, MA). All antibodies were used at a 1:1000 dilution. -actin antibody (#A2228) was obtained from Sigma-Aldrich (St. Louis, MO) and was used at a 1:10,000 dilution.

### Apoptosis Annexin V/PI Assay

Cells were collected and washed with cold 1XPBS followed by Annexin V and PI staining using the Alexa Flour 488 Annexin V/Dead Cell Apoptosis kit (Invitrogen) per the manufacturer’s recommendation. Flow cytometry analysis was performed using a FACS Calibur (BD Biosciences). Post-FACS analysis was performed using the Flow-Jo program (Tree Star, Ashland, OR).

### qRT-PCR

Trizol (Life Technologies, Grand Island, NY) was used to isolate total RNA from cells according to the manufacturer’s protocol. The extracted RNA was quantified using an ND-1000 spectrophotometer (Nanodrop, Wilmington, DE) and complementary single strand DNA (cDNA) was synthesized using an Omniscript RT kit (Qiagene, Valencia, CA). qRT-PCR experiments were performed as previously described [29].

### Statistical analysis

Student’s t-test was used to compare data from two groups, and one-way ANOVA with Tukey’s posthoc testing was used to evaluate the differences amongst multiple groups with p<0.05 considered as statistically significant.

### Transcriptome analysis

Using the stably transfected MHCC97-H c-Met shRNA and MHCC97-H scrambled shRNA cell lines, mRNA was extracted and hybridized to an Illumina human gene chip in biological triplicates according to the manufacturer’s protocol and as described [20]. QIAGEN’s Ingenuity Pathway Analysis (IPA, QIAGEN Redwood City, CA) was used to identify enriched pathways in MHCC97-H c-Met KD cells compared to scrambled shRNA control. The gene expression dataset is available at http://www.ncbi.nlm.nih.gov/geo (accession number GSE38343). Genes that had a statistically significant (p <0.05) 1.4-fold or greater change in expression between c-Met shRNA and scrambled shRNA cell lines were considered differentially expressed.

## Results

### EGFR/ErbB3 pathways are up-regulated after c-Met knockdown in c-Met constitutively activated HCC cells

**Fig 1:**
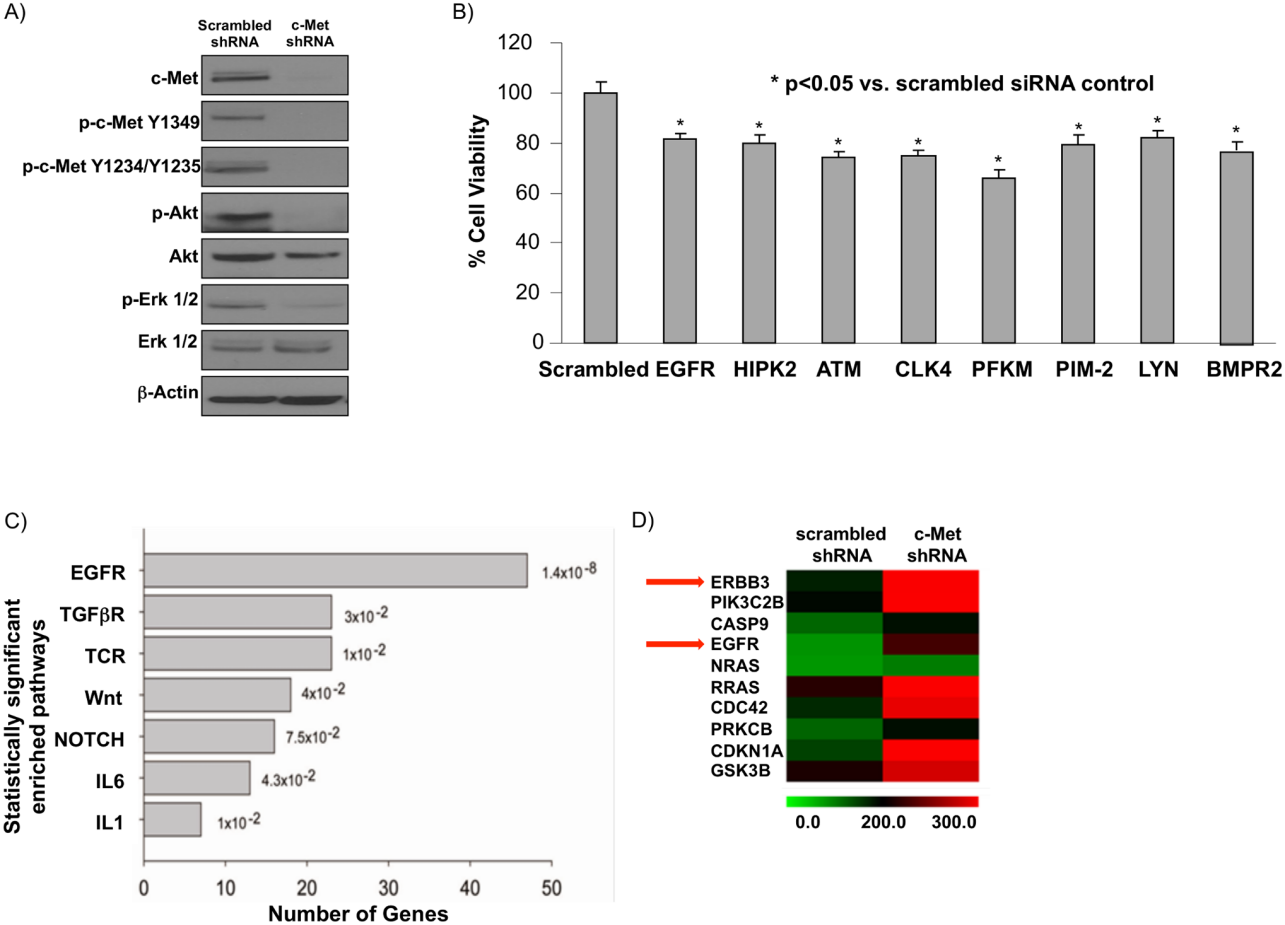
siRNA screening and microarray analysis of MHCC97-H liver cancer cell line stably transfected with c-Met shRNA reveals EGFR pathway as a putative survival pathway in HCC. A) c-Met shRNA was stably transfected into the MHCC97-H cell line, which has constitutive c-Met activity. After puromycin selection, immunoblot determined c-Met knockdown in a c-Met^+^ HCC cell line suppresses downstream signaling (c-Met, Akt, and Erk1/2 phosphorylation) compared to MHCC97-H cells stably expressing a scrambled shRNA. B) An XTT assay was performed to confirm the eight targets from the siRNA screen that had the greatest effect on cell viability in MHCC97-H c-Met KD cells. 10 nM siRNA and 0.2 ul RNAiMAX were used to transfect MHCC97-H c-Met KD cells and cell viability was determined at 48 hours post transfection. C) Ingenuity pathway analysis was conducted to compare microarray gene expression between MHCC97-H c-Met knockdown (KD) cells and MHCC97-H cells stably expressing a scrambled shRNA. The top seven enriched pathways are shown. D) A heatmap of the subset of the EGFR pathway gene set that is differentially expressed by microarray (Illumina human gene chip). A statistically significant (p <0.05) 1.4-fold or greater change in expression between c-Met shRNA and scrambled shRNA cell lines was considered differentially expressed.

In order to identify a putative bypass mechanism that is required for tumor survival after c-Met inhibition, we used c-Met shRNA to develop a stable c-Met knockdown (KD) cell line in the c-Met^+^ MHCC97-H cell line (referred to as MHCC97-H c-Met KD cells). Compared with scrambled shRNA transfected MHCC97-H (referred to as MHCC97-H shRNA control) cells, MHCC97-H c-Met KD cells have decreased c-Met expression with significantly suppressed phospho-c-Met (p-c-Met) and the downstream targets of the c-Met pathway, phospho-Akt (p-Akt) and phospho-Erk (p-Erk) (Fig 1A).

To further investigate the potential tumor survival mechanisms after c-Met knockdown in MHCC97-H cells, we conducted an siRNA library screen using 873 kinases and phosphatases in MHCC97-H c-Met KD cells. In 96-well plate format siRNA for each of the 873 targets was seeded into individual wells. Three scrambled siRNAs served as negative controls to obtain baseline cell viability. Potential survival pathways were determined by cell viability assay with a Z-score of -2 or less [30]. From the siRNA screen, we identified 17 potential targets including EGFR. To validate those targets, MHCC97-H c-Met KD cells were individually transfected with these potential target siRNAs, and then XTT cell viability assays were completed using eight technical replicates. Successful validation was defined as having a statistically significant suppressed cell viability (p<0.05) as compared to a scrambled siRNA control. Eight siRNA targets met these criteria (Fig 1B and S1 Table).

We next employed microarray analysis to determine gene expression changes representative of pathways maintaining cell survival in the absence of c-Met activity. We used Ingenuity Pathway Analysis (see Methods) to determine pathways that are enriched in MHCC97-H c-Met KD cells compared to shRNA control cells. Interestingly, the EGFR pathway contained the most enriched genes in MHCC97-H c-Met KD cells compared to shRNA control cells (Fig 1C). Furthermore, the subset of the EGFR gene set that was differentially expressed (defined as statistically significant 1.4-fold change in expression) in MHCC97-H c-Met KD cells compared to scrambled shRNA control cells contained EGFR family genes *ErbB3* and *EGFR* (*ErbB1*) (Fig 1D). Based on our siRNA screening analyses and microarray data, EGFR was selected for further investigation.

### EGFR is not a concomitant pathway for c-Met^+^ cell growth and survival

**Fig 2:**
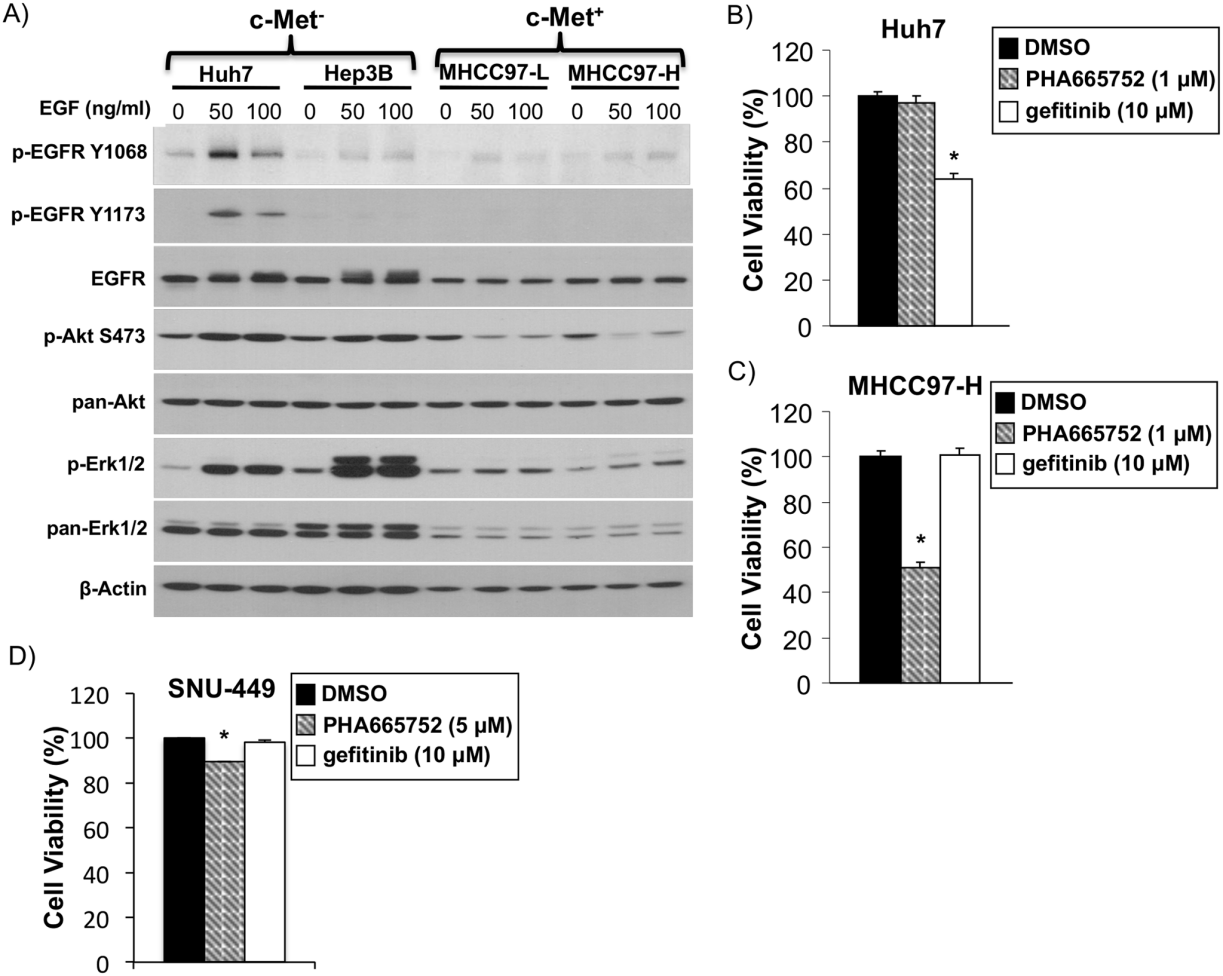
EGFR is a compensatory, not concomitant survival pathway in c-Met^+^ HCC. A) Immunoblot of c-Met^-^ cell lines Huh7 and Hep3B and c-Met^+^ cell lines MHCC97-L and MHCC97-H 24 hours post EGF treatment (0, 50, or 100 ng/ml) for EGFR, Akt and Erk signaling pathway activation. B) XTT cell viability assay of c-Met^-^cell line Huh7 treated with c-Met inhibitor PHA665752 (1 μM), EGFR inhibitor gefitinib (10 μM) or DMSO control 48 hours after treatment. C) XTT cell viability assay of c-Met^+^ MHCC97-H treated with c-Met inhibitor PHA665752 (1 μM), EGFR inhibitor gefitinib (10 μM) or DMSO control 48 hours after treatment. D) XTT cell viability assay of c-Met^+^ SNU-449 cell line treated with c-Met inhibitor PHA665752 (5 μM), EGFR inhibitor gefitinib (10 μM) or DMSO control for 48 hours. *statistically significant compared to DMSO control by Student t-test (p <0.05).

To determine whether the EGFR pathway was a concomitant or a compensatory pathway, we first analyzed phosphorylated EGFR (p-EGFR), an activated form of EGFR and its downstream targets phosphorylated Erk (p-Erk) and phosphorylated Akt (p-Akt), at baseline or upon EGF ligand stimulation in c-Met^-^ Huh7 and Hep3B cells and in c-Met^+^ MHCC97-L and MHCC97-H cells. The basal p-EGFR level is barely detectable in MHCC97-H cells compared with Huh7 cells, although there is baseline expression of EGFR protein itself. Additionally, EGF treatment at 50 and 100 ng/ml leads to increased p-EGFR, p-Akt, and p-Erk levels in Huh7 cells, whereas they do not lead to increased levels in c-Met^+^ MHCC97-H and MHCC97-L cells (Fig 2A). Additionally, the cell viability of Huh7 cells, which have high EGFR expression and activation upon EGF stimulation, is significantly reduced with the EGFR pathway inhibitor gefitinib but not with the c-Met pathway inhibitor PHA665752 (Fig 2B). Gefitinib does not show reduced viability on c-Met^+^ MHCC97-H cells, whereas c-Met inhibition with PHA665752 does lead to reduced viability (Fig 2C). The HCC cell line SNU-449 was previously identified as being c-Met^+^ [31]. Gefitinib does not show reduced viability on c-Met^+^ SNU-449 cells, whereas c-Met inhibition with PHA665752 leads to reduced viability (Fig 2D). These results suggest that EGFR is not a concomitant pathway for c-Met^+^ HCC cell growth and survival.

### Combination therapy with an EGFR pathway inhibitor provides additional benefit to c-Met inhibition alone *in vitro*

**Fig 3:**
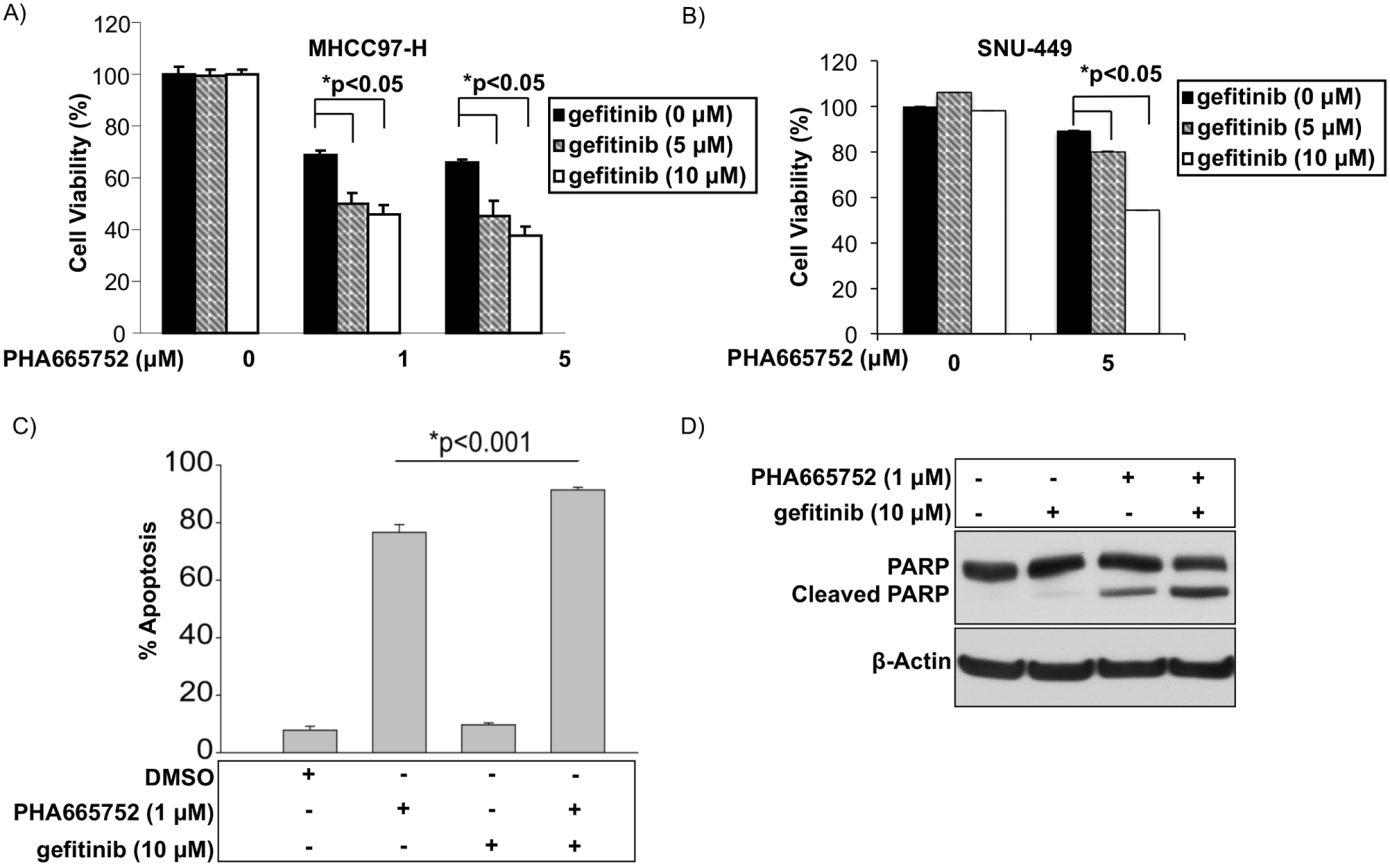
Combined inhibition of EGFR and c-Met in c-Met^+^ HCC leads to superior suppression of tumor growth than c-Met inhibitor alone in c-Met^+^ HCC. XTT cell viability assay 48 hours after treatment of A) MHCC97-H and B) SNU-449 cells treated with EGFR inhibitor gefitinib, c-Met inhibitor PHA665752 or both inhibitors. C) Apoptosis by flow cytometry of MHCC97-H cells treated with EGFR inhibitor gefitinib (10 μM), c-Met inhibitor PHA665752 (1 μM) or both. D) PARP cleavage by immunoblot 48 hours after treatment of MHCC97-H cells with EGFR inhibitor gefitinib (10 μM), c-Met inhibitor PHA665752 (1 μM) or both inhibitors.

Because EGFR was not found to be concomitantly active with c-Met in MHCC97-H and SNU-449 cells, we next explored the possibility that the EGFR pathway is induced as a compensatory survival pathway by the loss of c-Met activity. In order to test this hypothesis, we chemically suppressed the EGFR pathway simultaneously with c-Met pathway inhibition using the EGFR inhibitor gefitinib. Interestingly, compared to c-Met inhibition alone (PHA665752), c-Met inhibition in combination with gefitinib led to statistically significant decreases in cell viability in MHCC97-H cells (Fig 3A) and SNU-449 cells (Fig 3B). c-Met inhibition in combination with gefitinib led to increased apoptosis as determined by flow cytometry (Fig 3C) and apoptosis by immunoblot of PARP cleavage (Fig 3D). These results suggest that although EGFR is not active at baseline in c-Met^+^ cells, EGFR pathway members may be upregulated by c-Met inhibition.

### c-Met suppressed HCC upregulates EGFR pathway receptor ErbB3 and ligand TGF-α through an Akt-dependent survival mechanism

**Fig 4:**
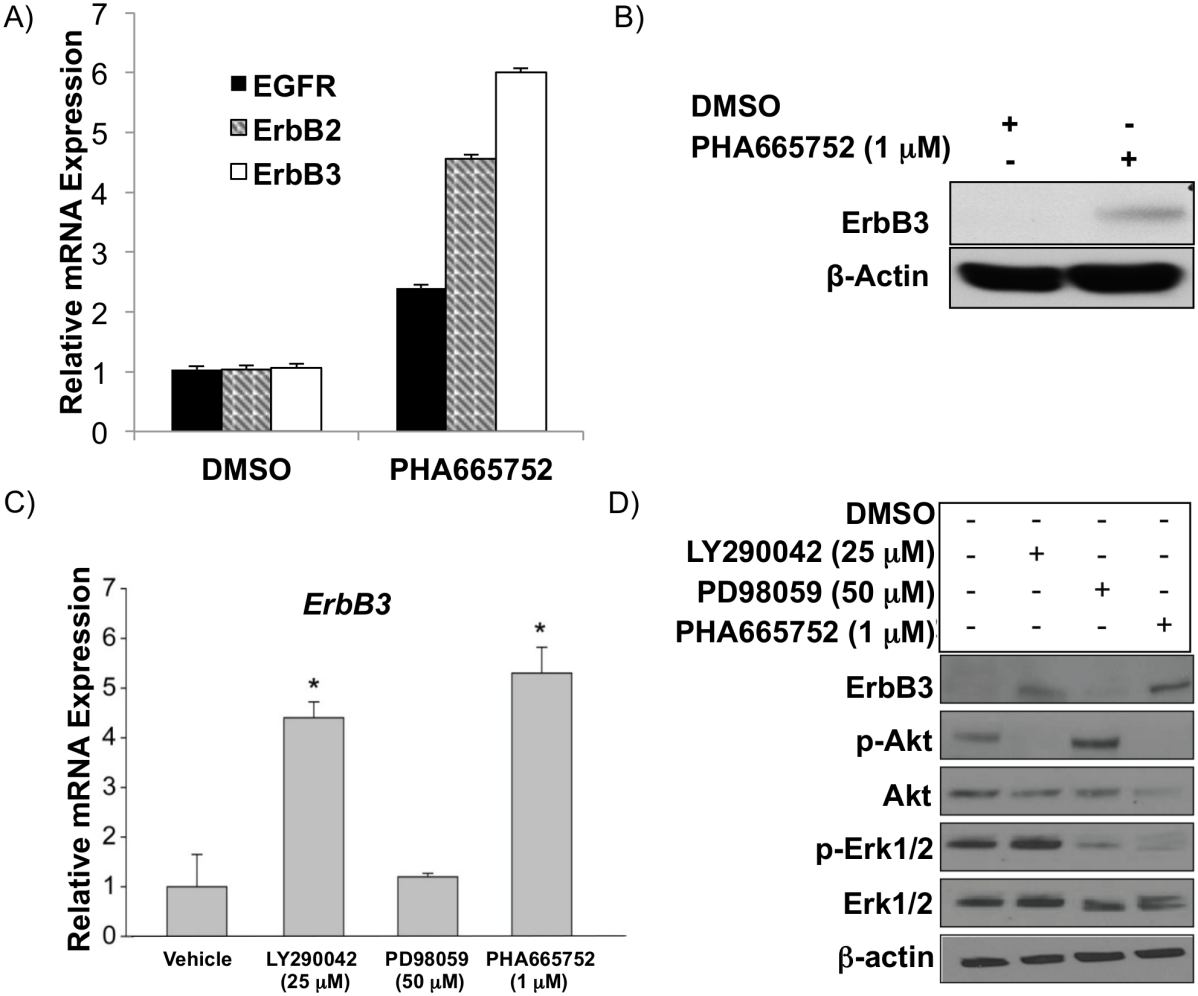
Suppression of c-Met in c-Met^+^ HCC upregulates ErbB3 predominantly through the PI3K/Akt signaling arm. (A) EGFR, ErbB2, and ErbB3 mRNA by qRT-PCR and (B) ErbB3 protein expression by immunoblot in MHCC97-H cells treated with c-Met inhibitor PHA665752 (1 μM) 48 hours after treatment. (C) ErbB3 mRNA by qRT-PCR and (D) protein by immunoblot in MHCC97-H cells treated with c-Met inhibitor PHA665752 (1 μM), PI3K inhibitor LY290042 (25 μM), or Mek inhibitor PD98059 (50 μM) 48 hours after treatment.

In order to test the hypothesis that the EGFR pathway is triggered as a compensatory mechanism for c-Met^+^ HCC survival after c-Met knockdown, we sought to determine which EGFR family members and/or ligands might be upregulated by c-Met inhibition. Our microarray data suggested that EGFR (ErbB1) and ErbB3 are upregulated by c-Met inhibition in MHCC97-H cells (Fig 1D). We sought to confirm whether EGFR and ErbB3 were upregulated after c-Met inhibition and also to determine whether the other EGFR family members (ErbB2 and ErbB4) were differentially expressed. We confirmed that EGFR and ErbB3 were upregulated after c-Met inhibition (1μM PHA665752) compared to vehicle control (DMSO) by qRT-PCR. We additionally saw that ErbB2 was up-regulated after c-Met inhibition; however, ErbB4 is undetectable both at baseline and after c-Met inhibition (Fig 4A). ErbB3 was detectable by immunoblot (Fig 4B) in MHCC97-H cells, which suggests that ErbB3 may play an important role in c-Met monotherapy resistance. Similarly, in SNU-449 cells EGFR, ErbB2, and ErbB3 were upregulated after c-Met inhibition (S1 Fig).

Because the c-Met pathway activates both PI3K/Akt and MAPK/Erk pathways [9–11], we next determined whether either of these downstream targets of c-Met signaling were specifically suppressing ErbB3 expression in the c-Met^+^ MHCC97-H cell line. We treated MHCC97-H cells individually with a PI3K inhibitor (LY290042; 25 μM), a Mek inhibitor (PD98059; 50 μM), or a c-Met inhibitor (PHA665752; 1 μM). We determined that LY290042 and PHA665752 led to statistically significant increases in ErbB3 mRNA (Fig 4C) and protein expression (Fig 4D) compared to vehicle control, whereas PD98059 did not significantly increase ErbB3 expression.

**Fig 5:**
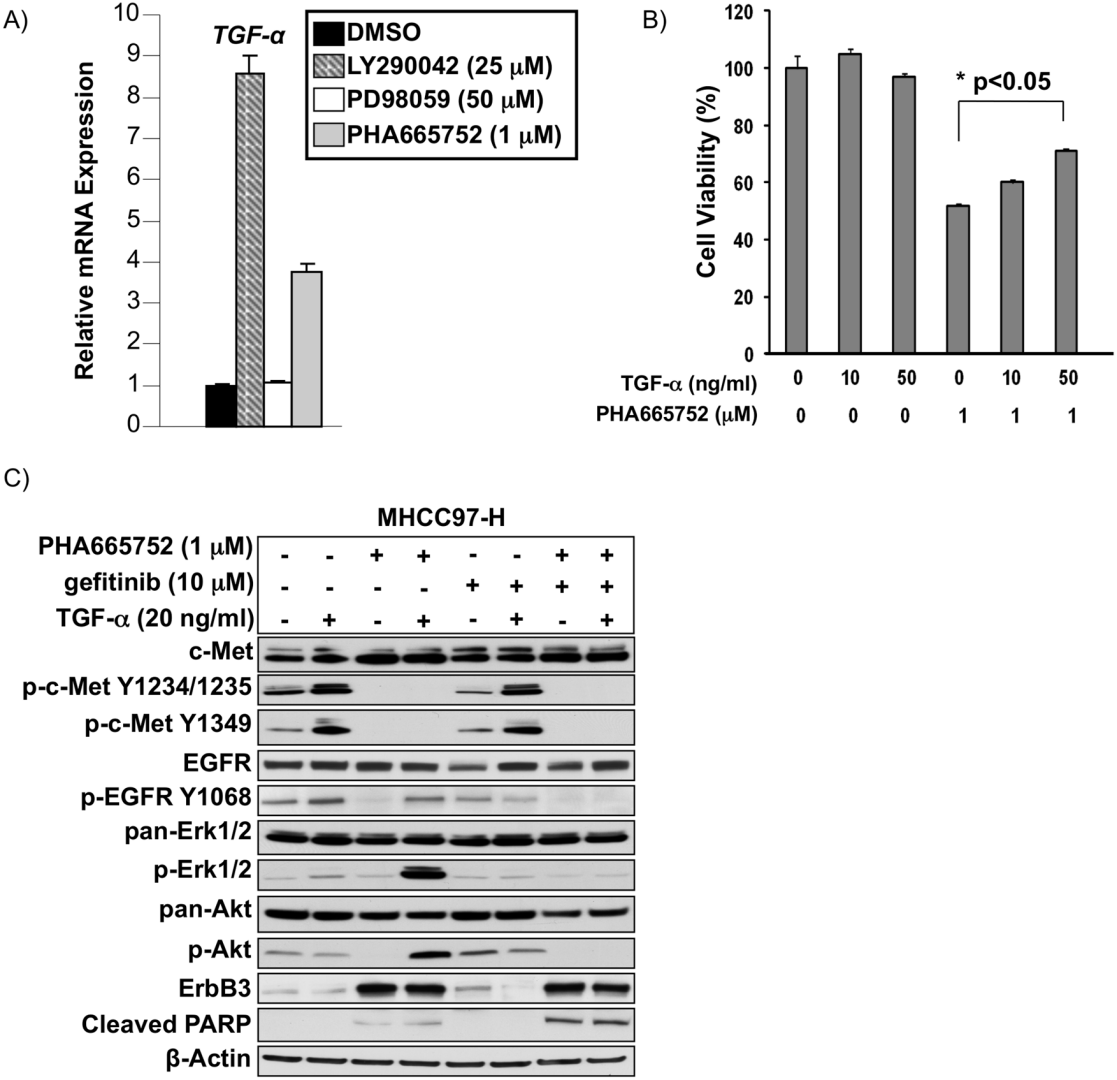
Transforming growth factor alpha (TGF-α), an EGFR ligand, is regulated by PI3K/Akt signaling downstream of c-Met and can act as a compensatory survival mechanism during c-Met blockade in c-Met^+^ HCC. A) TGF-α mRNA expression by qRT-PCR in MHCC97-H cells treated with c-Met inhibitor PHA665752 (1 μM), PI3K inhibitor LY290042 (25 μM), or Mek inhibitor PD98059 (50 μM) 48 hours after treatment. B) Cell viability by XTT assay of MHCC97-H cells treated with varying doses of TGF-α and/or c-Met inhibitor PHA665752 (1 μM) 48 hours after treatment. C) Immunoblot of MHCC97-H cells treated with combinations of TGF-α ng/ml), c-Met inhibitor PHA665752 (1 μM), or gefitinib (10 μM) for 48 hours. Immunoblot was performed for c-Met, p-c-Met, EGFR, p-EGFR, Akt, p-Akt, Erk1/2, p-Erk1/2, ErbB3, and cleaved PARP.

We performed a similar analysis of the ErbB ligand TGF-α and found it to be upregulated by treatment with 1 μM c-Met inhibitor PHA665752 or 25 μM of PI3K/Akt inhibitor LY290042 in c-Met^+^ MHCC97-H (Fig 5A). We further demonstrated that MHCC97-H cells pretreated with 1 μM PHA665752 had a dose-dependent increase in cell viability due to increasing doses of TGF-α treatment whereas vehicle-treated MHCC97-H cells did not (Fig 5B).

Immunoblot analysis revealed that compared to vehicle control, TGF- α can increase p-EGFR and p-Erk in MHCC97-H cells. c-Met inhibition by PHA665752 blocks c-Met phosphorylation, downstream Erk and Akt phosphorylation, and leads to increased cleaved PARP compared to vehicle control, while leading to increased ErbB3 expression. TGF-α treatment in the presence of PHA665752 leads to EGFR pathway activation as shown by increased p-EGFR levels, increased p-Erk, p-Akt, and ErbB3. The EGFR inhibitor gefitinib decreased p-EGFR but neither had an inhibitory effect on downstream targets p-Erk and p-Akt, nor increased PARP cleavage. Combination treatment with PHA665752 and gefitinib blocked c-Met and EGFR signaling and led to increased cleaved PARP compared to PHA665752 Fig 5C).

## Discussion

**Fig 6:**
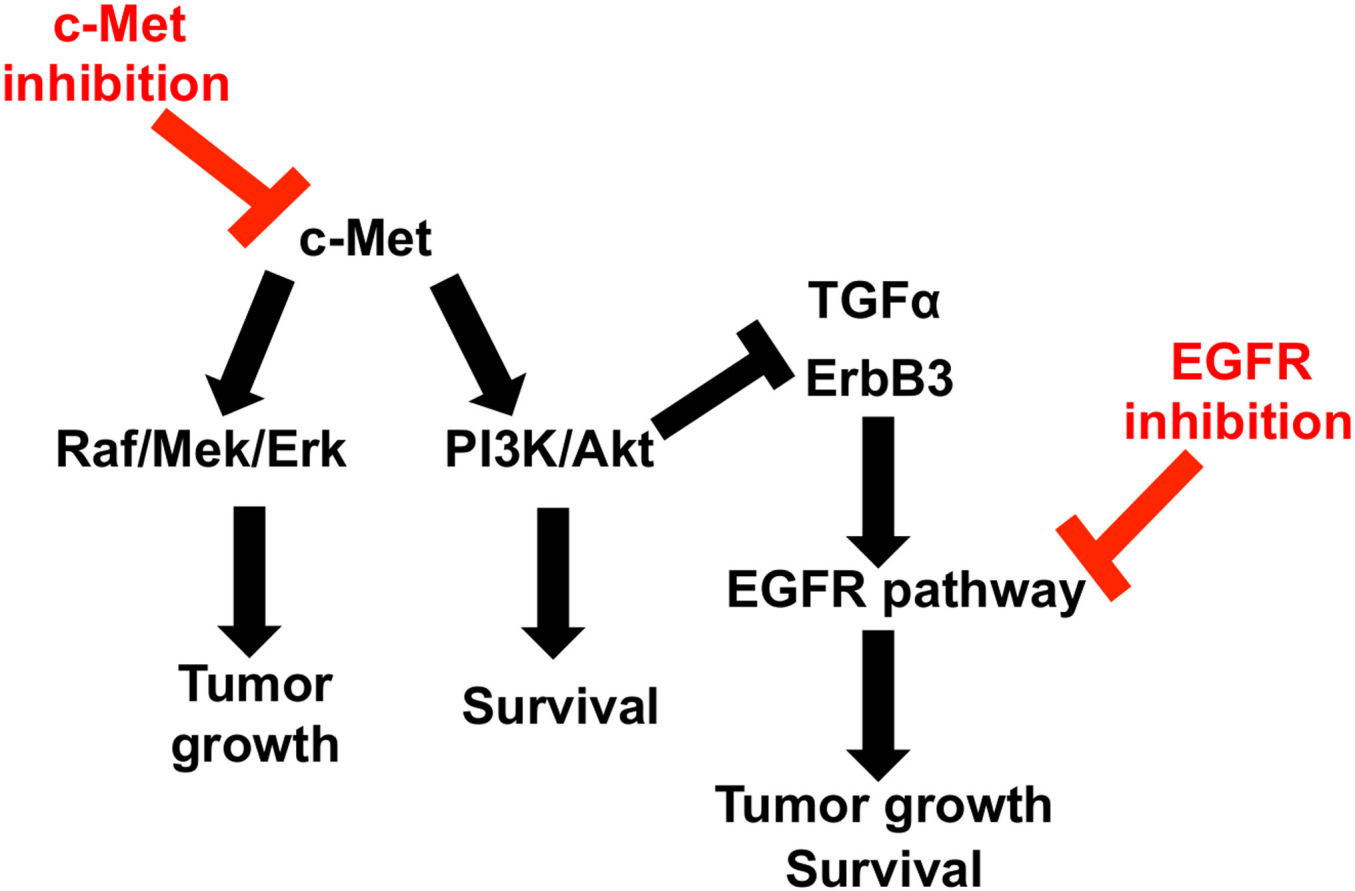
Schematic of c-Met and EGFR pathway crosstalk in c-Met^+^ HCC. c-Met activates MAP kinase (Raf/Mek/Erk) and PI3K/Akt signaling to induce HCC growth and survival. The PI3K/Akt arm of the c-Met signaling pathway normally suppresses EGFR pathway members (i.e. TGF-α and ErbB3), thus suppressing EGFR pathway activity. Suppression of c-Met signaling leads to loss of PI3K/Akt activity, and thus up-regulation of TGF-α and ErbB3 members of the EGFR signaling pathway. ErbB3 can heterodimerize with ErbB1 (EGFR), forming a potent EGFR receptor. Additionally, EGFR ligand TGF-α stimulates EGFR pathway activation, leading to cancer cell growth and survival. Targeting both EGFR and c-Met suppress pathway cross talk and leads to greater suppression of tumor growth and survival.

The HGF/c-Met oncogenic pathway is activated in approximately 50% of HCC, and expression levels of both HGF and c-Met are correlated with poor clinical outcomes in HCC [5–7, 32]. Currently, there are several c-Met inhibitors in clinical trials for multiple tumor types, including HCC. As described here and in our previous report, cells with constitutively active c-Met respond to c-Met inhibition; however, monotherapy does not completely eradicate tumor growth, indicating that a bypass tumor survival mechanism is likely involved in the maintenance of tumor growth in the presence of c-Met pathway suppression [21]. The goal of our study was to identify potential bypass mechanisms for tumor survival after c-Met suppression. Using siRNA screening and *in vitro* analysis, we identify that combination therapy with c-Met and EGFR inhibitors is superior to c-Met monotherapy *in vitro* (Fig 3). We further show that EGFR pathway activation is through up-regulation of ErbB3 and TNF-α in an Akt-dependent manner (Figs 4–6).

The EGFR (ErbB) family is a group of four structurally related receptor tyrosine kinases. This includes Her1 (EGFR, ErbB1), Her2 (Neu, ErbB2), Her3 (ErbB3), and Her4 (ErbB4). Evidence supports the four members of the ErbB protein family as capable of forming homodimers and heterodimers in order to activate downstream signaling cascades [33]. Additionally, there are eleven known growth factors that can activate specific ErbB family dimers. The EGFR pathway activates the MAPK/Erk and PI3K/Akt pathways leading to cell migration and proliferation [34].

Signaling interactions between c-Met and EGFR pathways have been reported in various tumor types but are incompletely understood. In non-small cell lung carcinoma (NSCLC), 70% of patients with Epidermal Growth Factor Receptor (EGFR) activating mutations will have a favorable initial response to EGFR inhibitors gefitinib or erlotinib [35]. However, the overwhelming majority of EGFR inhibitor responders will develop acquired resistance [36]. Interestingly, c-Met expression and activation have been associated with both primary and acquired resistance to EGFR inhibitor therapy in NSCLC patients [36–38]. The former is likely the result of c-Met and EGFR pathways being simultaneously activated in lung cancer, as inhibition of both pathways are required for maximal tumor reduction [39]. Regardless, studies suggest that c-Met may be an effective therapeutic target to overcome resistance to EGFR inhibitors in lung cancer [40]. Other studies in NSCLC suggest EGFR signaling through MAPK is sufficient to induce c-Met phosphorylation, leading to enhanced migration, invasion, and metastasis [41]. In other contexts, EGFR signaling can induce transcription of the *MET* gene, leading to higher c-Met expression in the cell membrane [42, 43]. More recently, it has been shown that EGFR and c-Met cross talk as well as gefitinib response are modulated by specific miRNAs [44, 45]. Induction of c-Met by EGFR inhibition has also been demonstrated in breast cancer and glioblastoma multiforme [46, 47]. These data support a strong link between the c-Met and EGFR pathways in lung, breast, and brain cancer.

We propose that similar dynamics are at play in HCC. However, whereas in previous studies EGFR pathway inhibition led to upregulation of the c-Met pathway or both pathways existed in parallel, we demonstrate that in c-Met^+^ HCC models, c-Met pathway inhibition leads to EGFR pathway upregulation. We found that c-Met is constitutively activated but that EGFR is not at baseline. In addition, monotherapy using an EGFR inhibitor has no significant effect on *in vitro* cell survival (Fig 2). However, c-Met inhibitor monotherapy triggered several survival mechanisms that bypass cell death caused by c-Met inhibitors through increased expression of EGFR ligand TGF-α and increased ErbB3 expression. We found that after blockade of c-Met using PHA665752, EGFR (ErbB1) is only slightly elevated by microarray (Fig 1D) and by qRT-PCR but not by western blot (data not shown), although EGFR is appreciably present at baseline in MHCC97-H cells (Fig 2A). Interestingly, it is well established that the EGF receptor family members can homodimerize and heterodimerize and that different dimers have different signaling potencies. ErbB3 can heterodimerize with ErbB1, forming one of the most potent signaling dimers [48]. Our data suggests that c-Met inhibition sensitized EGFR signaling through an increase in ErbB3 expression. Additionally, the expression of an EGFR ligand, TGF-α, suggests that an autocrine or paracrine mechanism may be involved in cancer cell survival after c-Met suppression, which requires further investigation.

Current clinical trials evaluating the efficacy of HGF/c-Met pathway inhibitors as monotherapy or in combination with other treatments are underway in patients with HCC and other solid tumors. In HCC, single agent c-Met inhibitors have shown modest effects. Foretinib, a multi tyrosine kinase inhibitor, was the first c-MET inhibitor to undergo clinical investigation in HCC and produced an overall response rate of 24% and median overall survival of 15.7 months in HCC patients never treated with sorafenib [49]. Tivantinib, a selective inhibitor of c-MET almost doubled median time to progression to 2.7 months from 1.4 months and median overall survival to 7.2 from 3.8 months in patients with c-Met-expressing tumors [50]. Clinical trials of c-Met inhibitors in combination with other therapeutics are currently underway in HCC and other solid tumors. Interestingly, combination therapy with c-Met and EGFR inhibitors in lung cancer are positive [51]. Our results suggest that combined c-Met and EGFR inhibitor therapy may be efficacious in HCC. Follow up *in vivo* pre-clinical studies are the focus of future work, and if successful, clinical trials are necessary to further determine the effect of combined suppression of EGFR and c-Met on HCC tumor growth.

## Supporting Information

S1 Fig Suppression of c-Met in the SNU-449 cell line upregulates EGF receptor family transcripts. c-Met^+^ SNU-449 cells were treated with PHA665752 or a DMSO for 48 hours. At 48 hours, RNA was harvested and expression of EGF receptor family members was measured by qRT-PCR. *represents statistical significance as determined by Student’s t-test (p<0.05).
(TIF)

S1 Table Genes validated as having a survival role in MHCC97-H KD HCC cell line. (DOCX)
