# Supplementary figures and images for: The EGFR/ErbB3 Pathway Acts as a Compensatory Survival Mechanism upon c-Met Inhibition in Human c-Met+ Hepatocellular Carcinoma
Source: PLoS One. 2015 May 22;10(5):e0128159. doi: 10.1371/journal.pone.0128159 (PMC4441360; doi:10.1371/journal.pone.0128159)

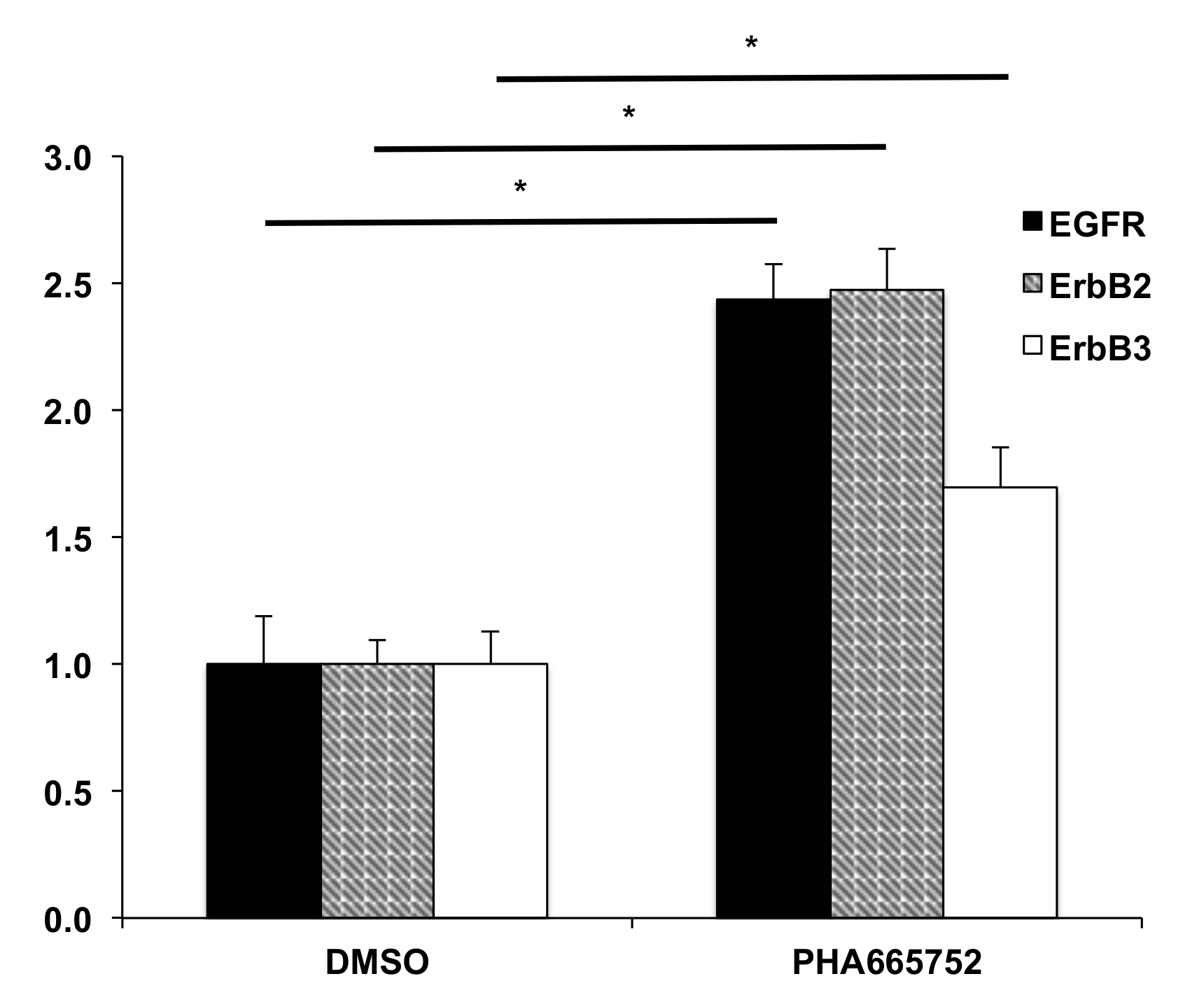

Supplement: S1 Fig — c-Met+ SNU-449 cells were treated with PHA665752 or a DMSO for 48 hours. At 48 hours, RNA was harvested and expression of EGF receptor family members was measured by qRT-PCR. *represents statistical significance as determined by Student’s t-test (p<0.05). (TIF) [file pone.0128159.s001.tif]
