# Supplementary material for: The EGFR/ErbB3 Pathway Acts as a Compensatory Survival Mechanism upon c-Met Inhibition in Human c-Met+ Hepatocellular Carcinoma
Source: PLoS One. 2015 May 22;10(5):e0128159. doi: 10.1371/journal.pone.0128159 (PMC4441360; doi:10.1371/journal.pone.0128159)
Supplement: S1 Table — (DOCX) [file pone.0128159.s002.docx]

| **Table S1: Genes validated as having a survival role in MHCC97-H KD HCC cell line** | |
| --- | --- |
| **Gene symbol** | **Full gene Name** |
| EGFR | epidermal growth factor receptor |
| HIPK2 | homeodomain interacting protein kinase 2 |
| ATM | Ataxia telaniectasia mutated |
| CLK4 | CDC-line kinase 4 |
| PFKM | phosphofructokinase muscle |
| PIM-2 | pim-2 oncogene |
| LYN | v-yes-1 Yamaguchi sarcoma viral related oncogene homolog |
| BMPR2 | bone morphogenitic protein receptor, type II |
